# Experimental Proof to Natural Hydrogen Generated Through Serpentinization of Olivine at Room Temperature and Ambient Pressure With Mechanochemistry

**DOI:** 10.1002/cssc.70850

**Published:** 2026-06-29

**Authors:** Jikai Ye, Derya Demirbas, Michael Felderhoff

**Affiliations:** ^1^ Department of Heterogeneous Catalysis Max‐Planck‐Institut für Kohlenforschung Mülheim an der Ruhr Germany; ^2^ Department of Molecular Theory and Spectroscopy (JWS) Max‐Planck‐Institut für Kohlenforschung Mülheim an der Ruhr Germany

**Keywords:** Mössbauer spectroscopy, mechanochemistry, olivine, serpentinization, white hydrogen

## Abstract

Natural hydrogen, or “white hydrogen,” is a promising low‐cost, low emission, yet abundant form of hydrogen. The redox reaction between water and iron(II) species in minerals such as olivine is considered the most important process (e.g., serpentinization) to generate natural hydrogen. Instead of at high temperatures or pressures, this work presents the possibility of the reaction via only mechanical energy input.

## Graphical abstract

This work shows experimentally the possibility of serpentinization (main mechanism of natural hydrogen) at room temperature and ambient pressure, which may provide the rapidly growing hydrogen economy with inspirations to explore new natural hydrogen deposits and new methods for hydrogen production.

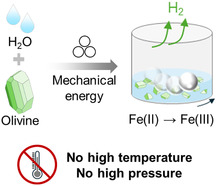

## 1 Introduction

Hydrogen is increasingly recognized as a key element in achieving a sustainable and carbon‐neutral economy. It has always played a significant role as a feedstock for chemical industry and has also shown great potential as a clean energy carrier owing to its high gravimetric energy density and carbon‐free nature [1]. Green hydrogen is considered the cleanest form of hydrogen, which is produced by electrolyzing water with electricity generated from renewable resources, achieving near‐zero emissions. It is therefore a promising candidate to replace fossil fuels for the decarbonization in sectors such as chemical industry, metallurgy, transportation, and energy storage. However, one of the major challenges for green hydrogen is its high production cost (€3–€7/kg H_2_), compared to gray hydrogen (€1–€2/kg H_2_), produced from fossil fuels without carbon capture, and blue hydrogen (€1.5–€3/kg H_2_), which incorporates carbon capture and storage [2, 8]. The expensive cost of green hydrogen mainly comes from the electrolyzer system as well as sustainable electricity [9, 10]. Numerous scientific and industrial efforts are made to lower its capital and operational expenditure [11, 13].

As an alternative source of hydrogen with low emissions, natural hydrogen, which is sometimes termed as white, gold, geologic, or native hydrogen, has attracted more attention in recent years [14, 15]. Natural hydrogen refers to hydrogen present naturally on earth. Its exploitation also has very low greenhouse gas intensity—currently even lower than blue or green hydrogen [3, 4, 8, 16]. Since natural hydrogen occurs naturally in subsurface environments and can be directly tapped with minimal processing, its production is much cheaper, with only about €0.5–€1/kg H_2_, making it a very cost‐effective alternative as low‐emission hydrogen [4, 5, 17]. Due to lack of awareness and difficulty of analysis, natural hydrogen has been neglected for many decades [14, 16, 18]. Moreover, drilling‐induced artifacts were often interfering with a solid conclusion about the widespread natural existence of hydrogen [19, 21]. This all led to the underestimation of natural hydrogen for many years. The assumed amount of hydrogen from geological sources has therefore been changing over time, with an estimation in 2020 proposing the annual flow of natural hydrogen to be 23 million metric tons (Mt, 10^9^ g) per year by analyzing available reports [18]. Another recent estimation used a stochastic model and predicted a total subsurface hydrogen resource of ~5.6 × 10^6^ Mt and an annual generation rate of 500 Mt [22]. The current global hydrogen demand is about 100 Mt, while hydrogen generated with low emissions accounts for only less than 1 Mt [4]. These predictions suggest that natural hydrogen has the potential to constitute a significant and previously underappreciated source within the global hydrogen market.

Natural hydrogen is believed to be generated through various mechanisms, including deep‐seated mantle or primordial degassing, water‐rock reactions, radiolysis of water, decomposition of organic matter, biological activities, and processes associated with volcanic/hydrothermal systems [15, 18, 23]. Among these pathways, water–rock reactions with iron‐bearing ores, such as serpentinization, and radiolysis of water from natural decay of radioactive elements are considered the two dominant sources of natural hydrogen, while serpentinization is by far the most investigated process [8, 15, 16]. Serpentinization involves the oxidation of ferrous iron (Fe(II)) in primary minerals (like olivine and pyroxenes) to ferric iron (Fe(III)), thus forming serpentinite and magnetite while reducing water to molecular hydrogen [8, 24, 25]. Previous experimental research was often conducted under high temperatures and/or high pressures, which is typical for deep geological environments at volcanic regions. Studies typically adopted temperatures up to 400°C, with the range between 200°C and 320°C being the best temperature for H_2_ generation via serpentinization due to optimized thermodynamics and reaction kinetics [26, 30]. Pressures ranging between 300 and 500 bar were often chosen to emulate conditions under deep crust [26, 28, 31], with some being kept as high as 5.5 kbar [29]. Nevertheless, harsh conditions are not necessities for the hydrogen generation process via serpentinization. Research confirms that H_2_ generation from oxidation of Fe(II)‐containing minerals is possible at lower temperatures or even room temperature (30°C–100°C), which, however, usually requires a very long experimental period (weeks to years) to show detectable hydrogen signals due to sluggish reaction kinetics [8, 24, 32, 33]. The long timescale may influence or even limit the prediction of the generation and existence of natural hydrogen.

Mechanochemistry describes the utilization of mechanical processing tools such as ball milling, twin‐screw extrusion, sonication, etc. to induce chemical and structural transformations [34, 35]. Many chemical reactions can be achieved under much milder conditions [36, 37]. Faults are often the center of interest for the exploration of geological gases, due to their potential contribution to the generation, promotion of migration, and structural trapping of the gases [38, 40]. By providing mechanical energy, methods like ball milling are commonly used in laboratory settings to experimentally emulate the mechanical fracturing (cataclasis) that occurs during seismic activities within natural fault zones [41, 44]. Many studies have found that through the rupture of chemical bonds (e.g., ≡Si─O─Si≡) of silicate minerals and thus the formation of reactive Si─O· radicals, hydrogen can be released from water, with a much faster rate even at much lower temperatures to room temperature and ambient pressure [41, 43, 48]. However, research on the mechanochemical interactions between water and olivine remains limited, with previous studies focusing on CO_2_ sequestration (carbonation) and hydrogenation within the CO_2_–(water)–olivine system [48, 53]. Furthermore, these investigations that observed hydrogen generation all employed stainless steel vessels and milling balls, which complicates the interpretation of hydrogen generation processes as well as the evolution of iron species, as the stainless steel milling system itself can react with water and release H_2_ [54]. It was also reported that power ultrasound could induce the redox reaction between olivine and water, yet such conditions are not likely to be available in nature [55].

In this work, we investigated the mechanochemical interactions between olivine and water, generating hydrogen at room temperature and ambient pressure. Ball milling was used to provide mechanical energy, and zirconia (ZrO_2_) was used as milling materials for the jar and balls to avoid redox interference. An inert atmosphere was employed to prevent reactions between olivine and CO_2_ and/or O_2_, which also reflects the conditions commonly present in the deep Earth. The generation of hydrogen as well as observable oxidation of iron species was confirmed, which provides more compelling evidence for the possibility of hydrogen generation through serpentinization under ambient conditions, driven solely by mechanical energy associated with tectonic movements.

## 2 Results and Discussion

**FIGURE 1:**
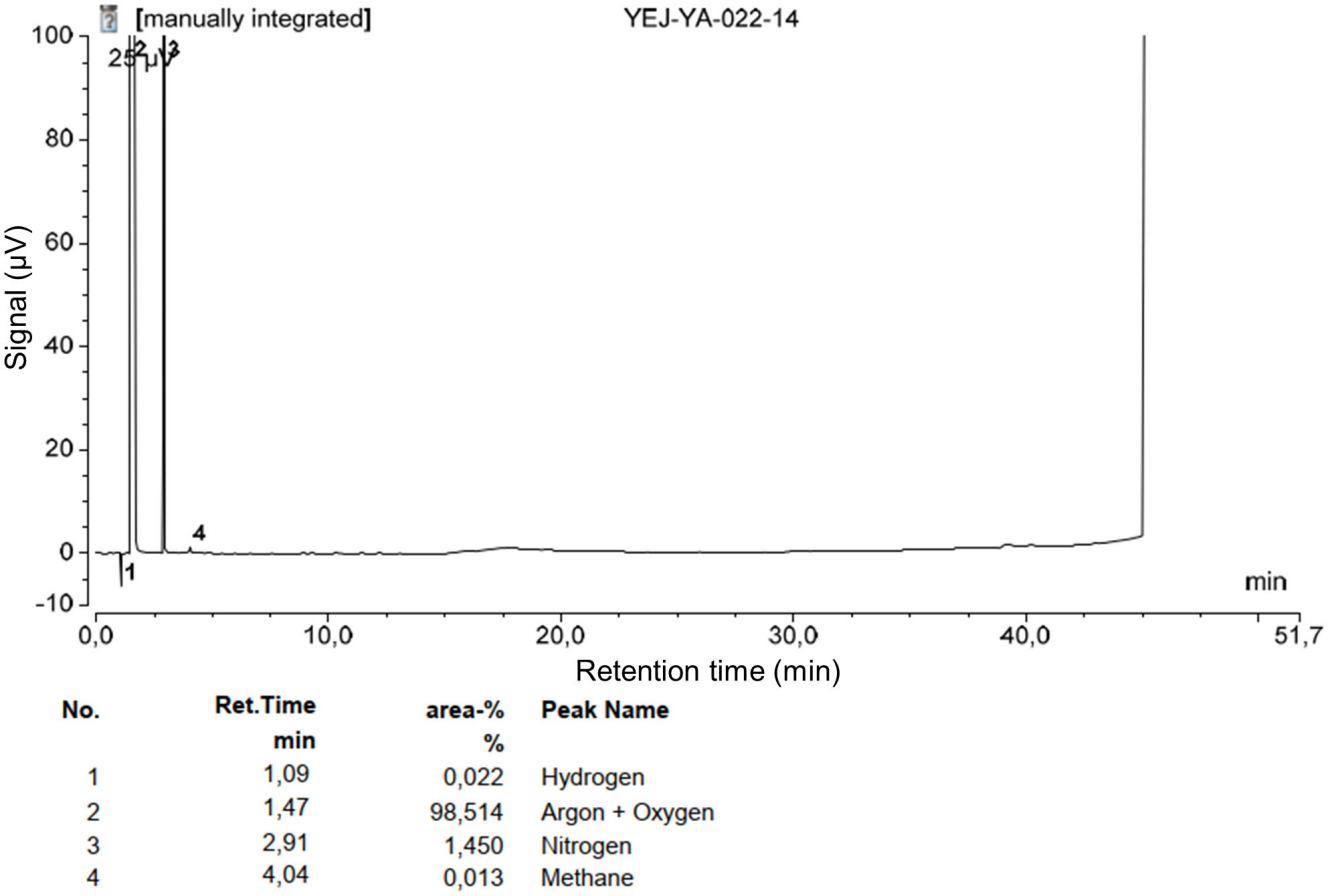
GC profile of the gas phase from olivine milled with water under argon.

To test out mechanochemical hydrogen generation from water, different materials were ball milled with water. ZrO_2_ milling balls and jar (Figure S1) were used to avoid the interference from the reactions between milling materials and water. Olivine is obtained in the form of small granules (2–4 mm), which is pre‐milled for 30 min before all experiments and measurements to have fine powder for easy handling, as shown in Figure S2. Ball milling was conducted under Ar to exclude the influence from CO_2_ and O_2_ in the air. After olivine was milled with water for 30 h, significant hydrogen signal was detected from the gas phase with gas chromatography (GC), as shown in Figure 1. Unlike most other gases, hydrogen has higher thermal conductivity than helium, which is the carrier gas for the measurements. The characteristic downwards‐pointing peak therefore signals hydrogen. Additionally, argon is the atmosphere where the reaction was performed. Oxygen and nitrogen come from air contamination, and possible methane sources might include partial decomposition of the polymer during milling [56, 57], such as the PEEK lid and/or the glue used to fix the zirconia inlet, as shown in Figure S1a; partial hydrogenation of any carbon species (impurities in olivine or contamination from the air) [37, 58], etc. Similar to the case at higher temperatures, the generation of hydrogen is likely the result of the redox reaction between water and Fe(II) species in olivine, where Fe(II) is oxidized to Fe(III) and water is reduced to hydrogen.

X‐ray diffraction (XRD) results of olivine before and after milling with water are shown in Figure S3. The natural olivine ore used in this work contains the structure of Mg_1.77_Fe_0.23_SiO_4_ and a small amount of Mg_2_(Si_2_O_6_). It is worth noting that for such a natural ore with complex composition, the structure derived from XRD only gives a hint, rather than the exact chemical composition. The serpentinization reaction between olivine and water likely took place during ball milling. A simplified equation is shown as Equation (1) with olivine of a similar composition [33].

(1) $${\mathrm{Mg}}_{1.8}{\mathrm{Fe}}_{0.2}{\mathrm{SiO}}_{4}+1.37H_{2}O\to 0.5{\mathrm{Mg}}_{3}{\mathrm{Si}}_{2}O_{5}{\left(\mathrm{OH}\right)}_{4}+0.3\text{Mg}{\left(\mathrm{OH}\right)}_{2}+0.067Fe_{3}O_{4}+0.07H_{2}$$

Fragmentation induced by the ball‐milling process can be observed under scanning electron microscope (SEM), as shown in Figure S4. Pre‐milled olivine typically has particle size in the micrometer range. After being milled with water, particles become much smaller, with a typical size of several tens to hundreds of nanometers. Grinded particles may reveal more reactive sites and thus promote reaction between water and iron species. The fragmentation also leads to reduced crystallite size [59, 60], which may explain the absence of observable new phase in the XRD pattern (Figure S3), except for peak broadening.

As a blank test, water was milled only with ZrO_2_ balls without any initial powder. Figure S5 shows the GC result of the gas phase after 30 h of milling. Different from when olivine was milled, the signal for hydrogen is barely visible, suggesting a small amount of hydrogen, which is likely the result of a similar mechanism as rock fracturing of SiO_2_ [41, 43]. As comparison, SiO_2_ was also milled with water, which gives a similar result (Figure S6) as the blank test. It is worth noting that in the blank test, without additional powder during milling, severe abrasion was observed. The XRD pattern of the abrasion is shown in Figure S7, which confirms the structure of the abraded material as mainly face‐centered cubic ZrO_2_ with a small part of monoclinic ZrO_2_.

Figure S8 shows the GC profile of the gas phase from olivine milling with water under air. Compared to that under argon, both the hydrogen and the methane signals appear weaker from milling under air. This may be partly due to the competitive oxidation of olivine with oxygen in the air, instead of generating hydrogen with water. The existence of oxygen has also been reported to suppress the generation of H_2_, for example, by reacting with radicals [44].

The temperature during ball milling was monitored, by measuring the exterior surface of the jar during milling olivine with water under argon (Figure S9) and during the blank test (Figure S10). In both cases, the temperature of the exterior surface of the jar increased to ~33°C from room temperature after approximately 4 h and remained constant within a narrow range. The interior temperature of the system may be presumably higher due to the insulation of the jar. To investigate the influence of temperature to the reaction between water and Fe(II) species in olivine, a control experiment was conducted by first milling olivine without water and then heating it with water. By milling without water, olivine is expected to be fragmented and more iron sites are likely revealed, similar to the case of being milled with water. As a conservative choice, the heating temperature was set to 80°C, which is supposedly higher than the temperature inside the milling jar during ball milling. This control experiment is set to investigate the possible mechanism where the redox reaction between Fe(II) and water could be mainly thermally driven, where ball milling merely helps activate the olivine via fragmentation of the particles. SEM image (Figure S11) confirms that the as‐treated particles are typically in the sub‐micro range while forming flake‐like surface microstructure, which is likely derived from partial dissolution and reprecipitation during thermal treatment of olivine with water [61]. The GC result of the gas phase shows significantly low hydrogen signal (Figure S12). This suggests that mechanical energy input, as is the case in tectonic movements, is itself a more efficient driven force for the reaction between water and iron‐containing rocks than high temperatures or high pressures, which were previously tested by many researchers under hydrothermal conditions [26, 62, 63]. Mechanically enhanced mass transfer also promotes good contact between Fe(II)‐species and water, mitigating Fe(II) partitioning, where ferrous iron is “hidden” in a secondary alteration mineral rather than being oxidized into magnetite [27].

**FIGURE 2:**
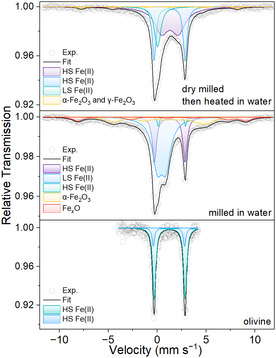
Mössbauer spectra of olivine powder after being pre‐milled, after being milled in water, and after being milled without water then heated in water.

Mössbauer spectroscopy enables a clear observation of the change of the oxidation states of iron species in olivine [64, 66]. All samples were recorded at 1.8 K. Measurements at 1.8 K are significantly more reliable, as the Lamb–Mössbauer factor reaches its maximum at low temperatures. At higher temperatures, fast magnetic relaxation can collapse sextets into doublets, complicating the identification of iron oxide phases. The spectra are shown in Figure 2 with parameters of the assigned sub‐spectra summarized in Table S1. Two Fe(II) doublets are observed in the Mössbauer spectrum of the pre‐milled olivine. The doublets are characterized by isomer shifts (*δ*) of approximately 1.21 and 1.29 mm s^−1^ and quadrupole splittings (Δ*E*_Q_) of approximately 3.16 and 3.20 mm s^−1^, respectively, which are characteristic for high‐spin Fe(II) in octahedral coordination. This confirms that all iron species in the olivine sample are in the +2 oxidation state. After ball milling with water, the sample shows HS Fe(II) center characterized by doublets with *δ* = 1.29 and 1.51 mm s^−1^, and Δ*E*_Q_ = 3.15 and 3.15 mm s^−1^, respectively. The sub‐spectrum with *δ* of 0.45 mm s^−1^ and Δ*E*_Q_ of 0.87 mm s^−1^ can be assigned to Fe(II) low‐spin. The changes in Fe(II) centers after ball milling are likely due to the lattice strain and partial de‐coordination induced by mechanical impact. Additionally, significant amount of Fe(III) appears characterized by sub‐spectra with *δ* = 0.49 and 0.50 mm s^−1^, Δ*E*_Q_ = 0.14 and 0.10 mm s^−1^, and hyperfine field (*H*) of 52.7 and 26.7 T, which can be assigned to Fe(III) species from *α*‐Fe_2_O_3_ and Fe_*x*_O, respectively. This suggests that fresh surface is revealed during milling induced by mechanical interaction, and some of the Fe(II) species are subsequently oxidized to Fe(III). Similarly, the sample first dry milled in argon and then heated with water also shows changes in Fe(II) centers after intensive milling. Sub‐spectra with *δ* = 1.34 and 1.28 mm s^−1^, and Δ*E*_Q_ = 1.72 and 3.17 mm s^−1^, respectively, can be assigned to high‐spin Fe(II). Sub‐spectrum with *δ* = 0.06 mm s^−1^ and Δ*E*_Q_ = 0.00 mm s^−1^ can be assigned to low‐spin Fe(II) with perfect symmetry. On the other hand, the contribution from Fe(III) is lower. The sub‐spectrum with *δ* = 0.28 mm s^−1^, Δ*E*_Q_ = 0.30 mm s^−1^, and *H* = 49.8 T can be assigned to a mixed state of maghemite *γ*‐Fe_2_O_3_ and hematite *α*‐Fe_2_O_3_. The area within the sub‐spectrum corresponding to Fe(III) species makes up only 4.5% of that within the whole spectrum, which is much lower than that in the sample milled with water, approximately 26.7%. This shows that more Fe(II) in olivine can be oxidized by mechanochemical reaction with water, thus suggesting that mechanical energy input is the main driven force for the redox reaction between olivine and water.

**FIGURE 3:**
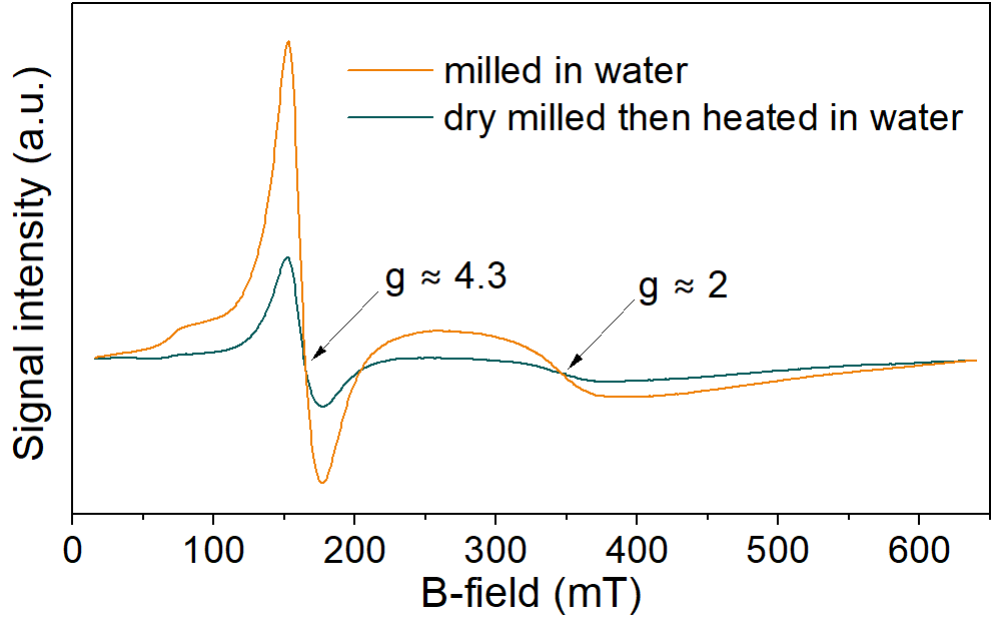
X‐band CW‐EPR spectra of olivine powder after being milled in water, and being heated in water after being milled without water.

X‐band continuous‐wave electron paramagnetic resonance (X‐band CW‐EPR) (Figure 3) results also agree with the observation. Both the sample milled with water and the sample dry milled in argon then heated in water exhibit characteristic signals at *g* ≈ 4.3 and 2. The signal at *g* ≈ 4.3 is attributed to rhombically distorted, weakly coupled high‐spin Fe(III) sites, typical for Fe^3+^ substituting in silicates [67]. The sample milled with water shows a substantially stronger *g* ≈ 4.3 signal than the sample first milled in argon and then heated with water, indicating a higher concentration of magnetically isolated or defect‐associated Fe(III) centers. The *g* ≈ 2 signal arises from magnetically coupled Fe(III) centers, typically associated with Fe_2_O_3_‐type clusters or nanoparticles. Both the line width and intensity of this signal increase with the strength of spin–spin coupling, making it a reliable indicator of the degree of magnetic ordering. The substantially higher *g* ≈ 2 signal intensity in the sample milled with water reflects a larger population of magnetically coupled Fe(III) centers, consistent with the formation of Fe_2_O_3_ clusters. The recorded spectrum extends beyond the accessible magnetic field range. Consequently, spin quantification based on double integration of the Fe(III) centers is unsuitable for a comparative analysis of the two samples. Notably, this enhanced magnetic coupling correlates directly with the significant hydrogen production observed for milling olivine with water.

**FIGURE 4:**
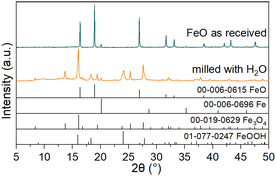
XRD patterns of FeO before and after milling with water under argon, with referential diffraction values of FeO (ICDD no. 00‐006‐0615), Fe (ICDD no. 00‐006‐0696), Fe_3_O_4_ (ICDD no. 00‐019‐0629), and FeOOH (ICDD no. 01‐077‐0247).

For a better observation of the mechanochemical reaction between Fe(II) and water, FeO was milled with H_2_O as a simplified scenario. GC result (Figure S13) suggests the existence of hydrogen after milling H_2_O with FeO, similar to the case with olivine. XRD result suggests the as‐received FeO is a mixture of FeO with a small amount of metallic Fe, as shown in Figure 4. After milling with water, it is oxidized to a mixture of Fe_3_O_4_ and FeOOH, where Fe(II) is partially oxidized to Fe(III).

## 3 Conclusion

In conclusion, we proposed a possible pathway for the generation of natural hydrogen at room temperature and ambient pressure, by emulating the mechanical energy input from tectonic movements with ball milling. Fe(II) species from olivine were partially oxidized to Fe(III) after milling with water upon observable hydrogen generation. The knowledge that natural hydrogen can be generated without high temperatures or high pressures, only with mechanical energy input, may inspire the discovery of new natural hydrogen deposits. New explorative actions must not be limited to regions with high pressure and/or high temperature, but also fault zones or regions rich in hydraulic energy. This research may also shed light on novel ways of utilizing mechanical energy with water‐involved redox reactions for hydrogen production purpose, such as the development of “orange hydrogen,” which is artificially stimulated natural hydrogen produced by injecting aqueous solution into reactive iron‐rich rock formations [25].

## Author Contributions

**Jikai Ye** **:** conceptualization, investigation, writing – original draft, methodology, visualization, writing – review and editing, data curation. **Derya Demirbas** **:** conceptualization, investigation, writing – original draft, writing – review and editing. **Michael Felderhoff** **:** conceptualization, investigation, writing – review and editing, methodology, supervision.

## Funding

This work was supported by the International Max Planck Research School for Sustainable Metallurgy (IMPRS SusMet).

## Conflicts of Interest

The authors declare no conflicts of interest.

## Supporting information

The authors have cited additional references within the Supporting Information [37, 56].

## Data Availability

The data that supports the findings of this study are available in the supplementary material of this article.
