# Supplementary material for: Experimental Proof to Natural Hydrogen Generated Through Serpentinization of Olivine at Room Temperature and Ambient Pressure With Mechanochemistry
Source: ChemSusChem. 2026 Jun 29;19(13):e70850. doi: 10.1002/cssc.70850 (PMC13313534; doi:10.1002/cssc.70850)
Supplement: Supplementary file 1 — The authors have cited additional references within the Supporting Information [37, 56]. [file CSSC-19-e70850-s001.pdf]

Supporting Information

# Experimental Proof to Natural Hydrogen Generated through Serpentinization of Olivine at Room Temperature and Ambient Pressure with Mechanochemistry

Jikai Ye[1], Derya Demirbas[2], Michael Felderhoff[1,*]

[1] Max-Planck-Institut für Kohlenforschung, Department of Heterogeneous Catalysis, Kaiser-Wilhelm-Platz 1, 45470 Mülheim an der Ruhr, Germany. E-mail: felderhoff@mpi-muelheim.mpg.de

[2] Max-Planck-Institute für Kohlenforschung, Department of Molecular Theory and Spectroscopy (JWS), Kaiser-Wilhelm-Platz 1, 45470 Mülheim an der Ruhr, Germany

## Table of Contents

**Experimental** .......... 2

**Material Characterizations** .......... 2

**Results and Discussions** .......... 3

**References** .......... 10

**Experimental**

Olivine is obtained from Donghai Xian Jiaxu Jewelry Co, Ltd., China, in the form of granules (2–4 mm).

Olivine ores were pre-milled for 30 min to obtain powder for the convenience of characterization with, for example, XRD, SEM, Mössbauer spectroscopy, and CW-EPR, as they generally require powder samples for easy sample preparation, and for a statistical characterization.

For the sample olivine milled with water: 1 g olivine, 2 mL distilled water and 20 g (6 × D10 mm) $ZrO_2$ (yttria-stabilized zirconia) balls were added into a $ZrO_2$ (yttria-stabilized zirconia) milling jar with a milling volume of ~41 mL and a total volume of ~45 mL. The air in the jar was replaced with Ar (1.3 bar) via Schlenk line for 3 times. A planetary ball mill (Pulverisette 6, Fritsch) was used for 30 h at a rotation speed of 500 rpm, with 10 min pause for every 60 min of milling to mitigate temperature increase of the system. Ball milling olivine with water under air was performed similarly, except that instead of replacing the atmosphere with Ar, an addition of ~0.3 bar synthetic air was charged into the system after assembling under air to have similar sampling conditions of the gas phase.

To have a simplified scenario for the observation of iron species, FeO in place of olivine was milled with water. Another test was done by replacing olivine with $SiO_2$. Blank test was done milling without any initial powders in the milling jar.

The change of temperature of the exterior surface the jar was recorded during ball milling with olivine with water under argon and the blank test for reference.

To exclude a thermally-driven reaction between olivine and water, 1 g olivine and 20 g (6 × D10 mm) $ZrO_2$ balls were first milled under Ar for 30 h at a rotation speed of 500 rpm, with 10 min pause for every 60 min of milling. The powder product was then transferred into an autoclave together with 2 mL $H_2O$. The atmosphere in the autoclave was replaced with Ar and the autoclave was kept at 80 °C for 30 h.

Before each milling session, milling balls with a mass lower than 90% (~3.0 g) of their original weight are replaced by new balls. All of the ball milling experiments were performed without heating.

**Material Characterizations**

The phase composition of powdery samples was characterized by X-ray diffraction (XRD, Stoe STADI P transmission diffractometer) with Mo $K_\alpha$ ($\lambda$ = 0.7093 Å). The instrument is equipped with a primary Ge(111) monochromator (Mo $K_{\alpha1}$) and a position-sensitive Mythen1K detector. Data were acquired in the range between 2θ of 5 and 50° with a step size of 0.015°. The measurement time was 20 seconds per step. Samples were filled into glass capillaries (D = 0.5 mm) for measurements.

Samples of the gas phase after milling were transferred via a gas bag (Supel™-Inert Multi-Layer Foil 0.6 L, Merck). The gas bag, the milling jar and a Schlenk line were connected via a three-way valve. The gas bag was cleaned by purging with Ar and evacuating to $10^{-3}$ mbar for 3 times. Afterwards, the jar was connected to the gas bag. Since the jar was originally charged with about 1.3 bar Argon before milling, some gas could be pushed from the jar to the gas bag once connected.

Gas chromatography (GC) equipped with a thermal conductivity detector (TCD) was used to analyze the gas-phase products after ball milling. Separation was carried out on a Rt-Molsieve 5A capillary column (28 m length, 0.53 mm inner diameter, 50 µm film thickness; column no. G/786). Helium was used as the carrier gas at a pressure of 0.5 bar. The injector and detector temperatures were maintained at 220 °C and 250 °C, respectively. The oven temperature program consisted of an initial isothermal step at 30 °C for 15 min, followed by heating at a rate of 8 °C $min^{-1}$ to 300 °C, with a final isothermal hold of 3 min. Gas samples (500 µL) were injected using a split ratio of 10:1. Although due to the limitations of the method, quantitative comparison of hydrogen generation was not possible, the relative results were highly reproducible.

Mössbauer spectra were recorded using a spectrometer operated in the conventional alternating constant-acceleration mode with a $^{57}Co$ source. The measurements at 2 K were achieved with a cryogen-free split-pair superconducting magnet system (Cryogenic Limited) equipped with an integrated variable temperature insert (VTI), enabling measurements over the temperature range of 1.5–150 K. The $^{57}Co$ source (1.8 GBq) embedded in a Rh matrix was kept at room temperature and placed at the zero-field position in the magnet gap using a re-entrant bore geometry. Isomer shifts are quoted relative to α-Fe foil (125 µm thickness) at 300 K. The minimum experimental line width, defined as the full width at half-maximum, was 0.24 mm $s^{-1}$. Mössbauer spectra were analyzed by simulation and using the MF2 program developed by Dr. Eckhard Bill, using the usual nuclear Hamiltonian.

Continuous wave X-band Electron paramagnetic resonance (CW-EPR) spectra were collected on a Bruker E500 ELEXSYS spectrometer system equipped with an ER4116DM dual-mode cavity and an Oxford Instruments ESR 900 continuous-flow liquid helium cryostat interfaced with an ITC Mercury temperature controller (3.8–300 K range). The microwave unit was a high-sensitivity ER049X Bruker superX bridge with integrated microwave frequency counter. EPR measurements were conducted at temperature 5 K, 20 mW microwave power, 100 kHz modulation frequency and 7.45 G modulation amplitude. The spectra were analyzed using eview programs developed by Dr. Eckhard Bill.

**Results and Discussions**

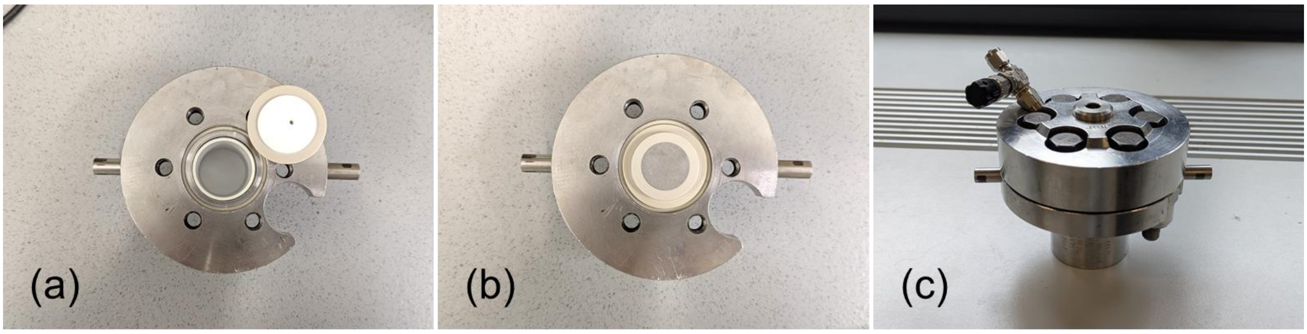


**Fig.S1** Photos of the $ZrO_2$ milling jar use in this work. (a) Opened jar with lid put aside. (b) Jar with lid put in place. (c) Jar closed with steel cover.

The milling jar is built by the Fine Mechanics Workshop of Max-Planck-Institut für Kohlenforschung. For more details of the milling jar, readers are referred to our previous work.[37] Epoxy resin glue is used to fix a $ZrO_2$ inlet (D = 33 mm, h = 48 mm) inside the stainless-steel milling jar. A lid is made by embedding $ZrO_2$ plate and a steel filter in a polyether ether ketone (PEEK) frame to avoid powder spillage. A steel cover enables atmosphere-adjustable experiments up to 200 bar.

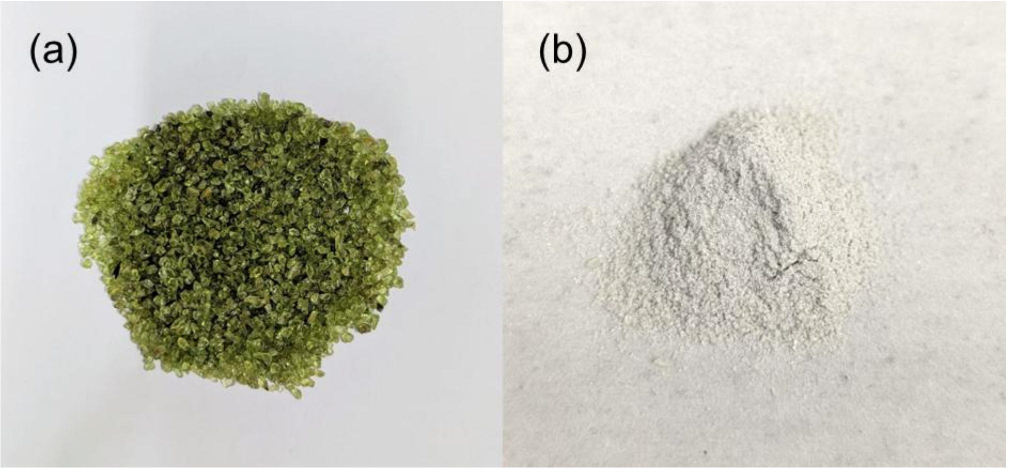


**Fig.S2** Photos of the (a) as-obtained olivine and (b) after being pre-milled for 30 min. All experiments and measurements in this work were conducted with the pre-milled powder.

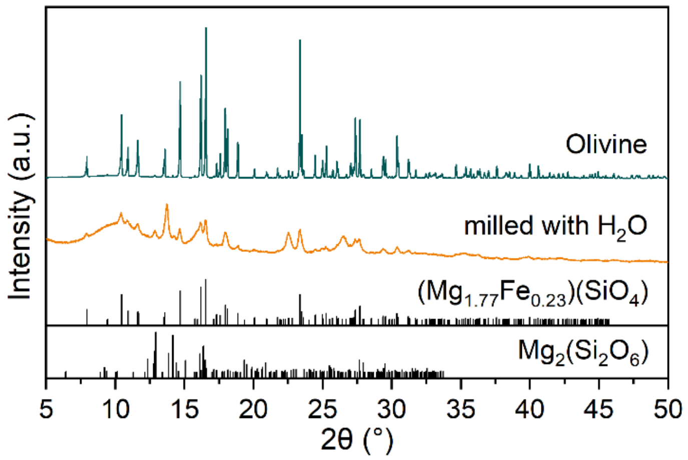


**Fig.S3** XRD patterns of olivine before and after milling with water, with referential diffraction values of $Mg_{1.77}Fe_{0.23}SiO_4$ (ICDD No. 01-070-7343) and $Mg_2(Si_2O_6)$ (ICDD No. 01-086-0430).

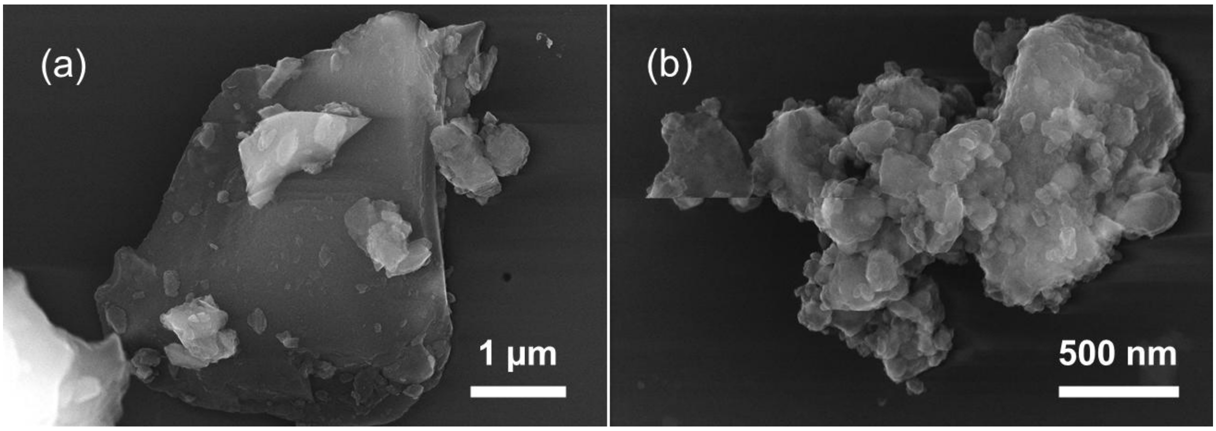


**Fig.S4** SEM images of typical particles of olivine after being (a) pre-milled, (b) milled with water under argon.

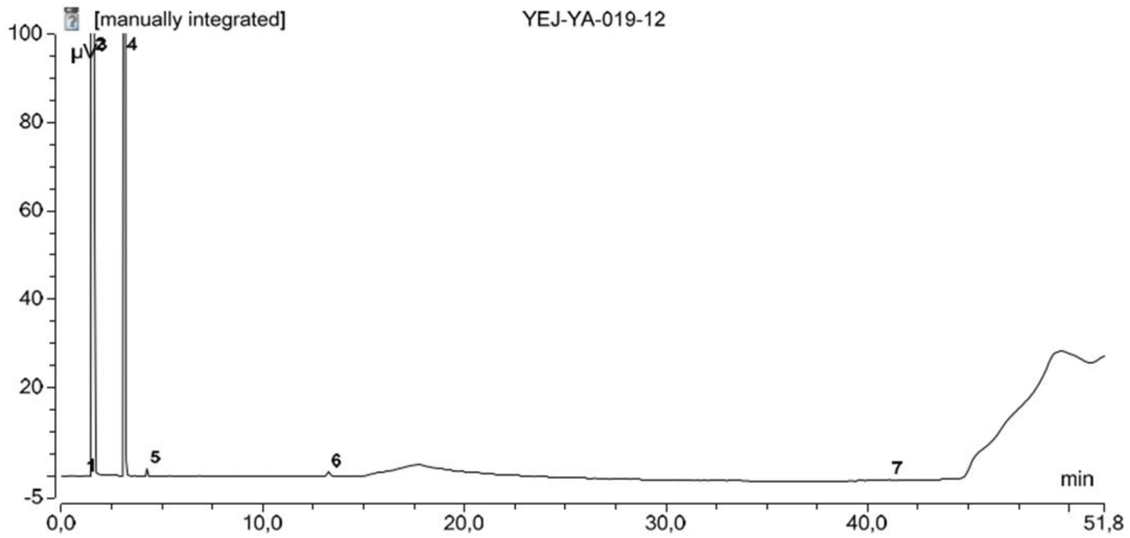


| No. | Ret.Time min | area-% % | Peak Name |
|---|---|---|---|
| 1 | 1,11 | 0,001 | Hydrogen |
| 2 | 1,50 | 93,277 | Argon (no baseline separation) |
| 3 | 1,63 | 0,662 | Oxygen (no baseline separation) |
| 4 | 3,13 | 5,994 | Nitrogen |
| 5 | 4,25 | 0,021 | Methane |
| 6 | 13,26 | 0,040 | |
| 7 | 41,09 | 0,005 | |

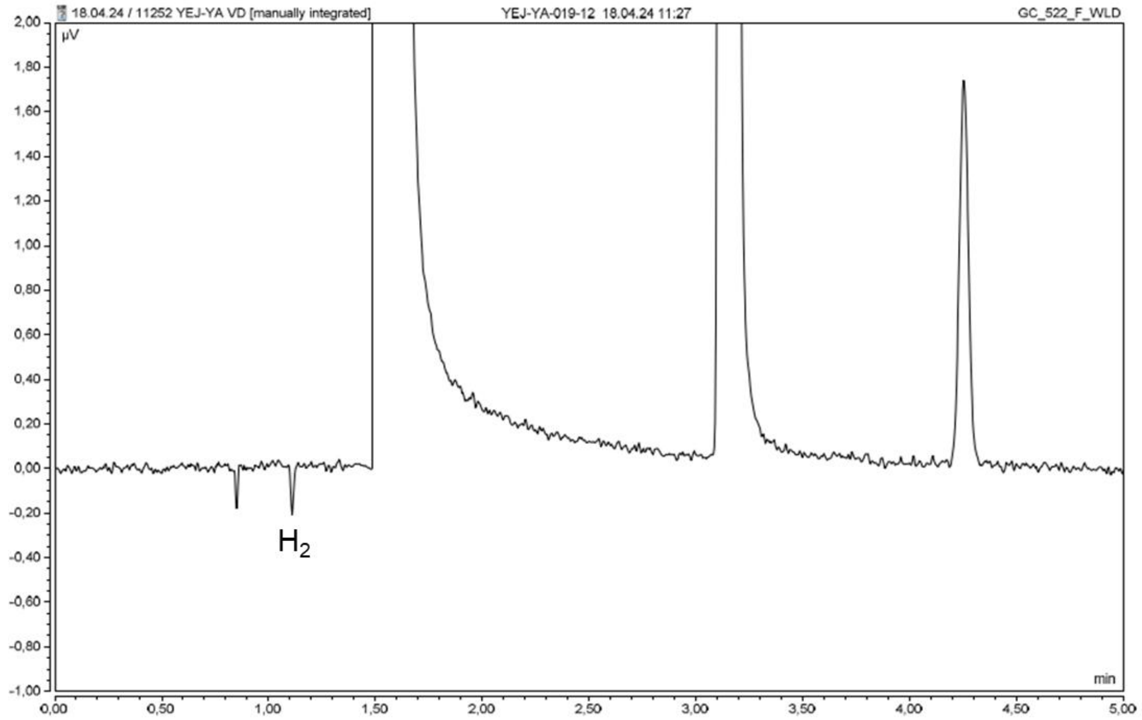


**Fig.S5** GC profile of the gas phase from milling water under argon in an empty $ZrO_2$ jar, with an enlarged section in the interest of the hydrogen.

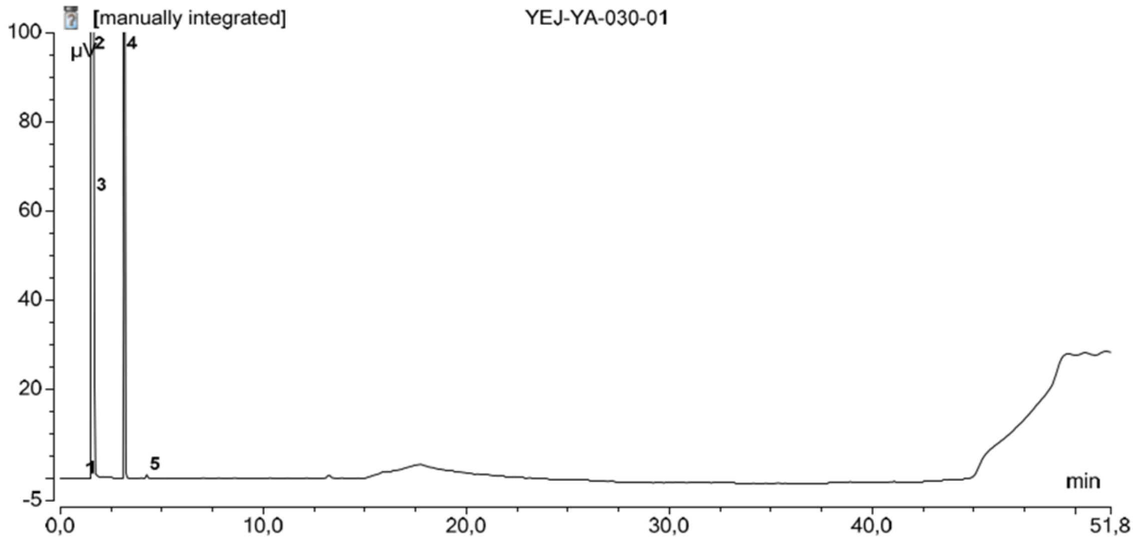


| No. | Ret.Time min | area-% % | Peak Name |
|---|---|---|---|
| 2 | 1,50 | 97,126 | Argon (no baseline separation) |
| 3 | 1,62 | 0,394 | Oxygen (no baseline separation) |
| 4 | 3,14 | 2,428 | Nitrogen |
| 5 | 4,24 | 0,008 | Methane |
| 6 | 13,22 | 0,029 | |
| 7 | 38,89 | 0,008 | |
| 8 | 41,08 | 0,006 | |

1 peaks out of 8 (total area percentage= 0,00 % ) are below threshold.

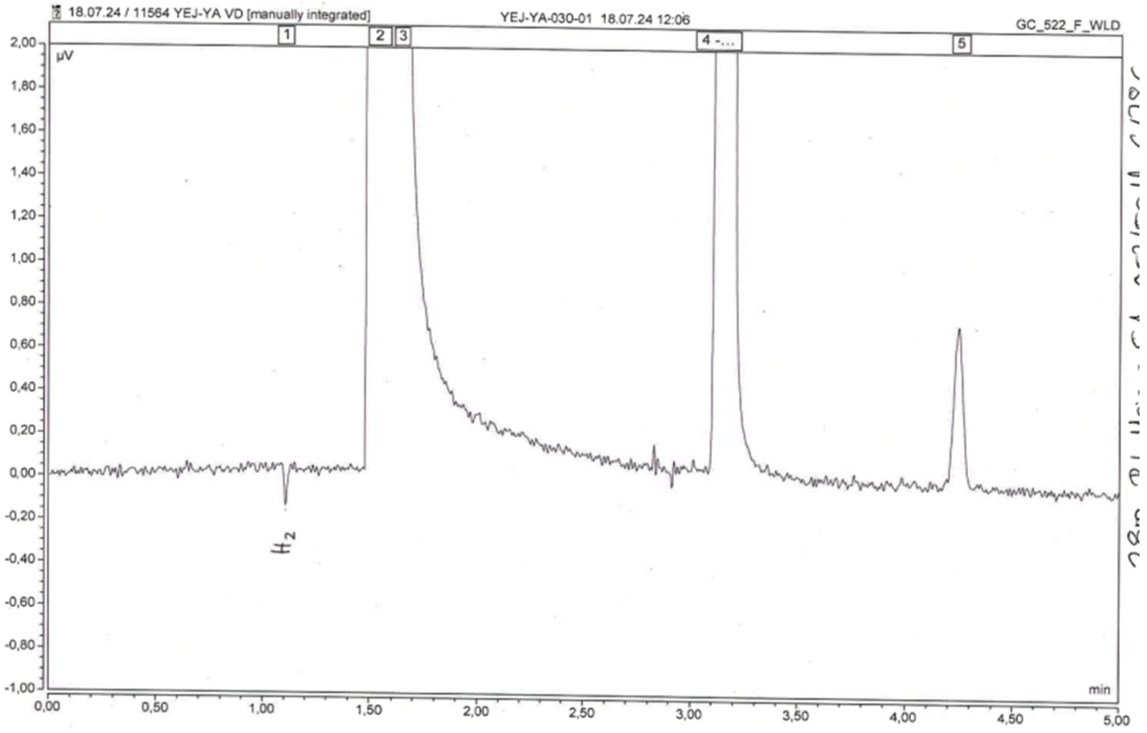


**Fig.S6** GC profile of the gas phase from $SiO_2$ milled with water under argon, with an enlarged section in the interest of the hydrogen.

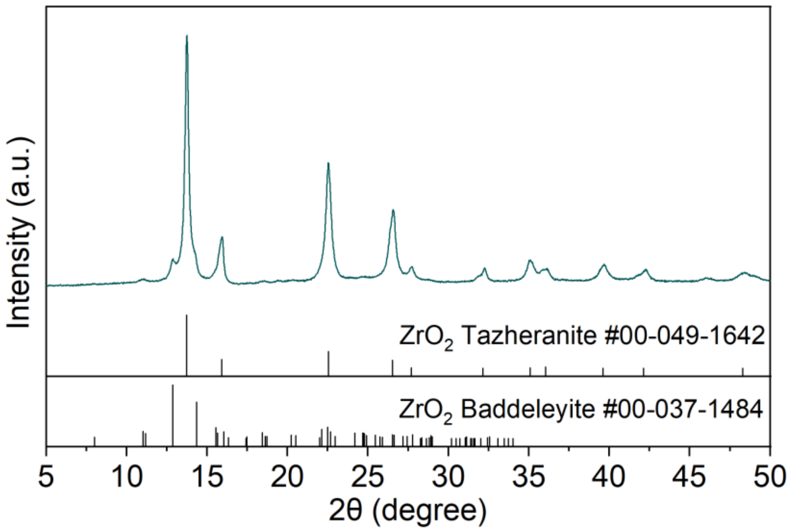


**Fig.S7** XRD pattern of the powders from abraded $ZrO_2$ jar and balls after milling with water, with referential diffraction values of face-centered cubic $ZrO_2$ (Tazheranite, ICDD No. 00-049-1642) and monoclinic $ZrO_2$ (Baddeleyite, ICDD No. 00-037-1484).

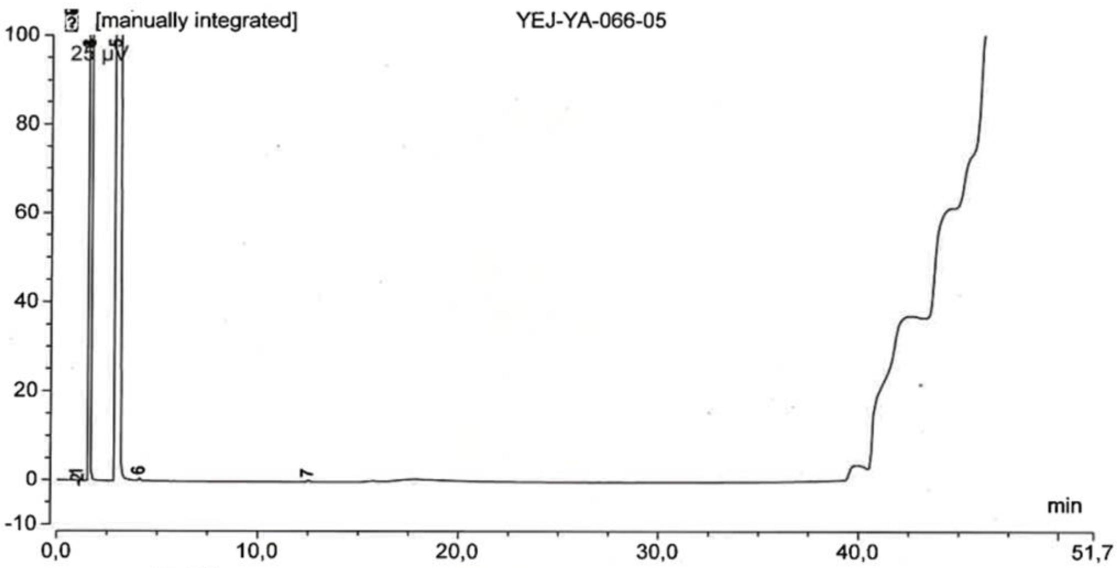


| No. | Ret.Time min | area-% % | Peak Name |
|---|---|---|---|
| 1 | 1,03 | 0,001 | |
| 2 | 1,09 | 0,004 | Hydrogen |
| 3 | 1,56 | 0,485 | Argon (no baseline separation) |
| 4 | 1,58 | 14,512 | Oxygen (no baseline separation) |
| 5 | 2,84 | 84,976 | Nitrogen |
| 6 | 4,12 | 0,009 | Methane |
| 7 | 12,51 | 0,014 | |

**Fig.S8** GC profile of the gas phase from olivine milled with water under air.

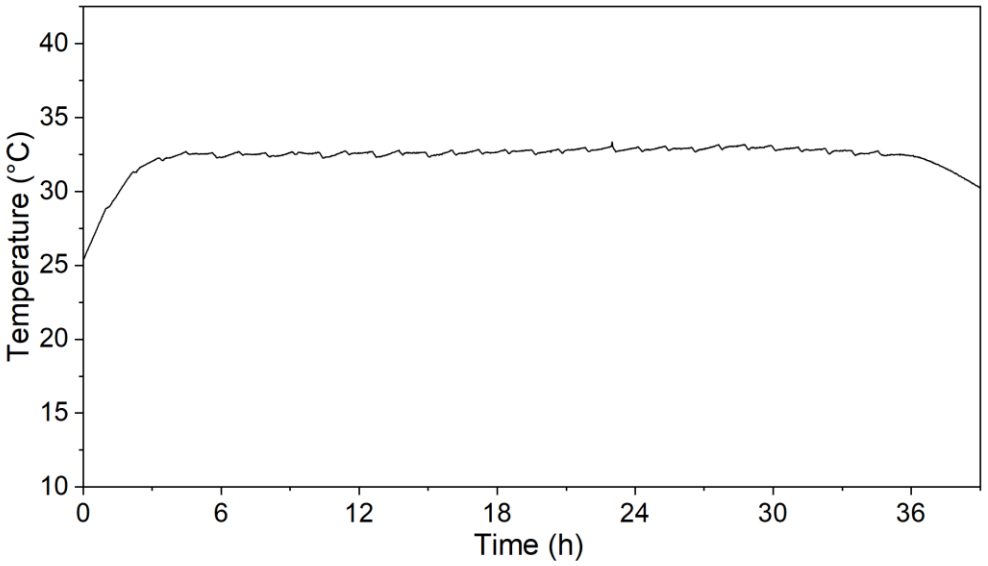


**Fig.S9** Temperature profile of the exterior surface of the milling jar during milling olivine with water under Ar.

The temperature is recorded by a thermocouple in contact with the exterior surface of the jar during milling. For details of temperature-recording set-up, readers are referred to our previous work.[56]

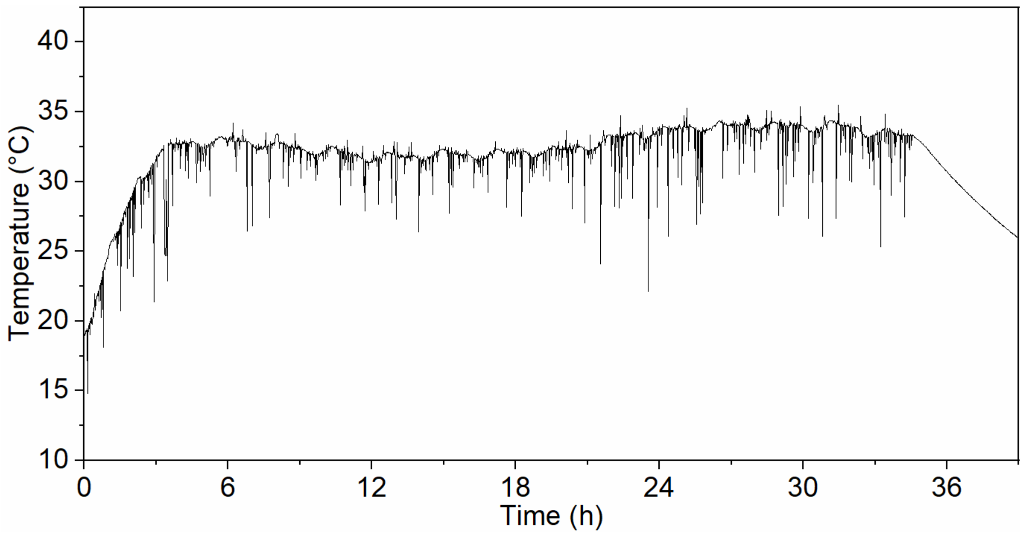


**Fig.S10** Temperature profile of the exterior surface of the milling jar during milling only $ZrO_2$ balls with water under Ar.

Spikes appear due to fluctuation of RF signals during milling when battery is low.

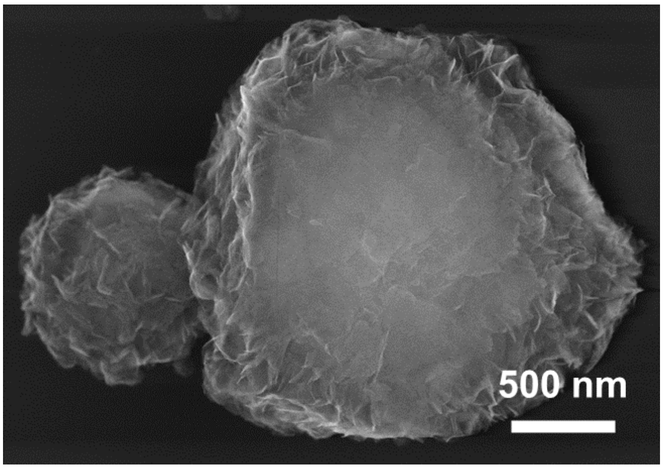


**Fig.S11** SEM image of typical particles of olivine after being milled without water then heated to 80 °C with water.

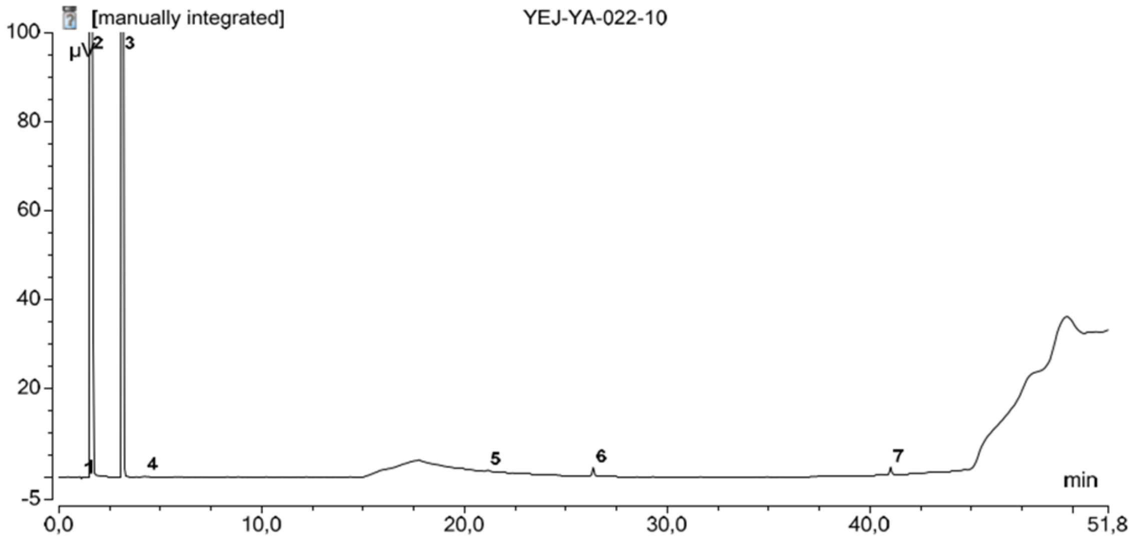


| No. | Ret.Time min | area-% % | Peak Name |
|---|---|---|---|
| 1 | 1,10 | 0,002 | Hydrogen |
| 2 | 1,50 | 91,192 | Argon + Oxygen |
| 3 | 3,08 | 8,703 | Nitrogen |
| 4 | 4,21 | 0,004 | Methane |
| 5 | 21,12 | 0,010 | |
| 6 | 26,35 | 0,050 | |
| 7 | 41,02 | 0,039 | |

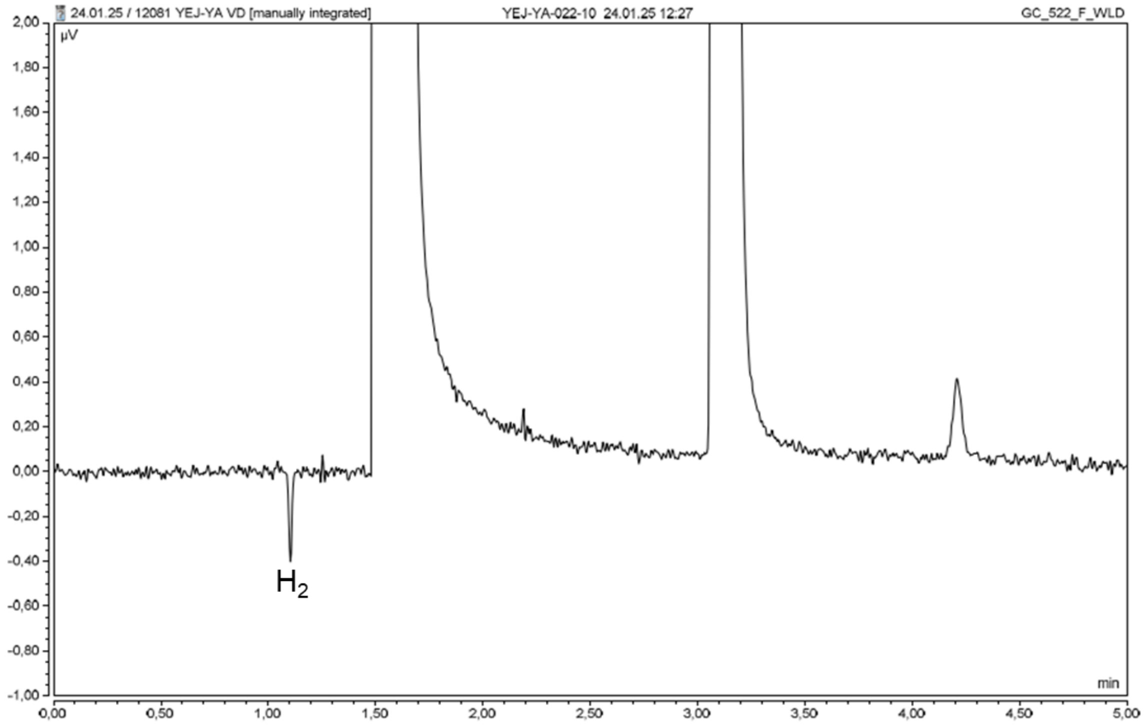


**Fig.S12** GC profile of the gas phase from olivine milled without water under Ar for 30 h, and then heated to 80 °C with water under Ar for 30 h, with an enlarged section in the interest of the hydrogen.

**Table S1** Parameters of the sub-spectra in the Mössbauer results in Figure 2.

| Sample | Sub-spectra | δ (mm s $^{-1}$) | $\Delta E_Q$ (mm s $^{-1}$) | H (T) | Rel. area (%) |
|---|---|---|---|---|---|
| Pre-milled olivine | HS Fe(II) | 1.29 | 3.16 | | 81.7 |
| | HS Fe(II) | 1.21 | 3.20 | | 18.3 |
| Milled in water | HS Fe(II) | 1.29 | 3.15 | | 29.2 |
| | LS Fe(II) | 0.45 | 0.87 | | 40.1 |
| | HS Fe(II) | 1.51 | 3.15 | | 19.6 |
| | HS Fe(III)/α-$Fe_2O_3$ | 0.49 | 0.14 | 52.7 | 10.1 |
| | $Fe_xO$ ($Fe^{3+}$) | 0.50 | 0.10 | 26.7 | 16.6 |
| Heated in water after milling without water | HS Fe(II) | 1.34 | 1.72 | | 46.9 |
| | HS Fe(II) | 1.28 | 3.17 | | 29.9 |
| | LS Fe(II) | 0.06 | 0.00 | | 18.7 |
| | HS Fe(III)/ mixed state of α-$Fe_2O_3$ and γ-$Fe_2O_3$ | 0.28 | 0.30 | 49.8 | 4.5 |

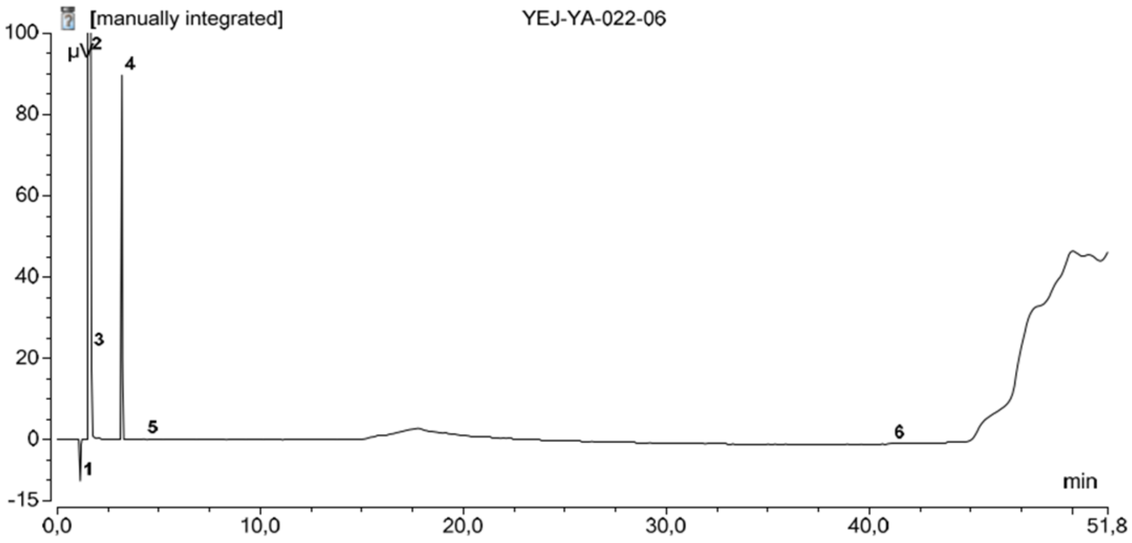


| No. | Ret.Time min | area-% % | Peak Name |
|---|---|---|---|
| 1 | 1,12 | 0,039 | Hydrogen |
| 2 | 1,51 | 98,926 | Argon (no baseline separation) |
| 3 | 1,64 | 0,178 | Oxygen (no baseline separation) |
| 4 | 3,18 | 0,846 | Nitrogen |
| 5 | 4,28 | 0,004 | Methane |
| 6 | 41,08 | 0,006 | |

**Fig.S13** GC profile of the gas phase from FeO milled with water under argon.

## References

[37] S. Reichle, M. Felderhoff, F. Schüth, *Mechanocatalytic Room-Temperature Synthesis of Ammonia from Its Elements Down to Atmospheric Pressure, Angew. Chem. Int. Ed.* **2021**, *60*, 26385-26389.

[56] L. Li, O. Vozniuk, Z. Cao, P. Losch, M. Felderhoff, F. Schüth, *Hydrogenation of different carbon substrates into light hydrocarbons by ball milling, Nat. Commun.* **2023**, *14*, 5257.
